# 186. Evaluating Clinical Outcomes for Treatment of Staphylococcal Bloodstream Infection in Patients with Febrile Neutropenia

**DOI:** 10.1093/ofid/ofab466.388

**Published:** 2021-12-04

**Authors:** Muneerah M Aleissa, Isabel H Gonzalez-Bocco, David W Kubiak, Sara Zekery-Saad, Jessie Signorelli, Sarah P Hammond, Jennifer Manne-Goehler, Francisco M Marty

**Affiliations:** 1 Brigham and Women’s Hospital, Boston, Massachusetts; 2 Massachusetts General Hospital, Boston, Massachusetts; 3 Massachusetts General Hospital, Dana-Farber Cancer Institute, Harvard Medical School, Boston, Massachusetts

## Abstract

**Background:**

*Staphylococcus aureus* bloodstream infections (BSIs) in patients with febrile neutropenia (FN) is associated with a mortality rate of up to 49%. For documented infections in patients with FN, guidelines recommend narrowing therapy once susceptibilities result and fever has resolved. Although anti-staphylococcal beta-lactams are the mainstay of treatment for Methicillin-Susceptible and Penicillin-Susceptible *Staphylococcus aureus* (MSSA and PSSA) BSIs, some clinicians opt to continue broad antibiotics against *Pseudomonas* during FN. Studies evaluating treatment modalities and outcomes of MSSA and PSSA BSI in patients with FN are lacking.

**Methods:**

We conducted a retrospective cohort study of adult patients with MSSA or PSSA BSI who received antibiotics for the treatment of FN (absolute neutrophil count < 500 cells/L and temperature > 100.4F) at Brigham and Women’s Hospital and Dana-Farber Cancer Institute from 1/2010 to 4/2021. Patients who received < 72-h of antibiotics were excluded. The primary outcome was composite clinical failure (60-day all-cause mortality and/or 60-day BSI recurrence). Other outcomes included inpatient mortality, 60-day readmission, 60-day infection outcomes, incidence of acute kidney injury and hepatotoxicity. Data was analyzed using Chi-Square test or Fisher’s Exact test.

**Results:**

Among 108 patients who met our criteria, 58% were male, median age was 57 years (IQR 44, 66), 94% had a hematologic malignancy, 4% had a solid tumor, and 2% had both. A total of 41 (38%) received combination therapy with broad spectrum and anti-staphylococcal beta-lactam, 48 (44%) received broad spectrum beta-lactam followed by anti-staphylococcal beta-lactam after neutrophil recovery, and 19 (18%) were narrowed to an anti-staphylococcal beta-lactam prior to resolution of neutropenia. Clinical failure was similar across all treatment arms (34% for combination therapy, 25% for broad spectrum beta-lactam, and 37% for anti-staphylococcal beta-lactam) (Table).

Table. Outcomes

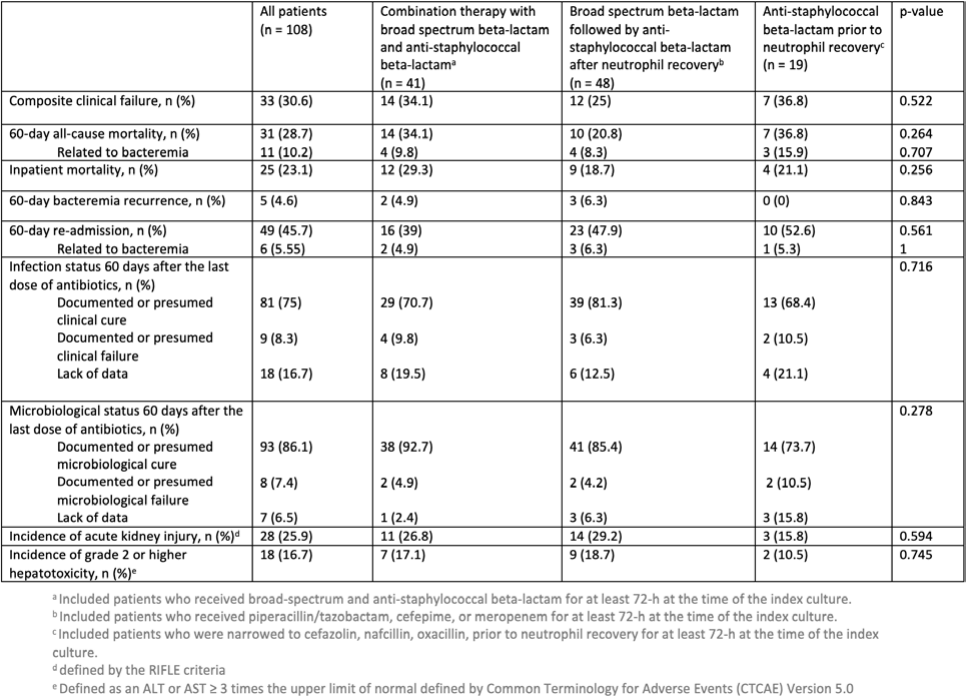

**Conclusion:**

De-escalation to an anti-staphylococcal beta-lactam prior to neutrophil recovery in FN patients with MSSA or PSSA BSIs did not result in significantly higher clinical failures. Further prospective studies are needed to support antimicrobial stewardship initiatives in oncology patients.

**Disclosures:**

**All Authors**: No reported disclosures

